# A Polyol-Mediated Fluoride Ions Slow-Releasing Strategy for the Phase-Controlled Synthesis of Photofunctional Mesocrystals

**DOI:** 10.3390/nano9010028

**Published:** 2018-12-26

**Authors:** Xianghong He, Yaheng Zhang, Yu Fu, Ning Lian, Zhongchun Li

**Affiliations:** School of Chemistry and Environmental Engineering, Jiangsu University of Technology, Changzhou 213001, Jiangsu, China; Zhangyaheng@jsut.edu.cn (Y.Z.); fuyu@jsut.edu.cn (Y.F.); ln@jstu.edu.cn (N.L.); lizc@jstu.edu.cn (Z.L.)

**Keywords:** polyol-assisted fluoride ions slow-release strategy, NaYF_4_ mesocrystals, crystallographic phase control

## Abstract

There are only a few inorganic compounds that have evoked as much interest as sodium yttrium fluoride (NaYF_4_). Its extensive applications in various fields, including transparent displays, luminescence coding, data storage, as well as biological imaging, demand the precise tuning of the crystal phase. Controlling the emergence of the desired α-phase has so far remained a formidable challenge, especially via a simple procedure. Herein, we represented a polyol-assisted fluoride ions slow-release strategy for the rational control of pure cubic phase NaYF_4_ mesocrystals. The combination of fluorine-containing ionic liquid as a fluoride source and the existence of a polyalcohol as the reactive medium ensure the formation of uniform α-phase mesocrystallines in spite of a higher temperature and/or higher doping level.

## 1. Introduction

Since inorganic micro/nanocrystals usually exist in various forms or phases, the phase transformation from kinetically stable ones to thermally stable ones is a normal phenomenon [1,2,3,4]. The intrinsic properties of a micro/nanomaterial are largely determined by its unique crystal structure [5,6]. Hence, controlling the phase formation is essential for both scientific interests and extended applications. As a typical example, sodium yttrium fluoride (NaYF_4_) owns two polymorphs under ambient condition, i.e., the cubic (α-) and hexagonal (β-) phase, which is a commonly used matrix lattice for up-conversion luminescence. The former is a high-temperature metastable phase, while the latter remains thermodynamically stable [7,8]. The past decades have witnessed much exploration of its controlled synthesis and up-/down-conversion luminescent properties [1,4,9,10,11,12,13,14,15,16,17,18,19,20,21,22,23,24,25,26,27,28,29,30,31,32,33,34]. Compared with considerable work on α→β phase transformation [1,4,9,10,11,12,13,14,15,16,17,18,19], the fabrication of α-NaYF_4_ as well as the investigation involving the β→α transformation process have been neglected [26,30,33,34]. So far, some strategies have been developed to fabricate α-phase NaYF_4_ nano-/mocro-crystal, such as a liquid–solid–solution (LSS) procedure [21], polyol method [22], two-phase interfacial route [23], microwave-assisted ionic liquid (IL)-based technique [24], modified solvothermal approach [25], and self-sacrificing template multiple-step route [26,27]. Furthermore, introducing Mn^2+^ (*r* = 81 pm) with a smaller size than Y^3+^ (*r* = 89 pm) into an NaYF_4_ host can dominate, forming pure α-phase NaYF_4_ nanoparticles [28]. However, α-phase NaYF_4_ inevitably transforms into the hexagonal ones due to its thermodynamic instability. Additionally, the cubic NaYF_4_ nanoparticles are usually formed preferentially in the solution system of non-equilibrium reactions [20]. As a consequence, rationally controlling α-NaYF_4_ and simultaneously avoiding the generation of β-phase or a mixture of α and β phases remain formidable challenges, especially via a simple procedure [28,29,34]. 

On the other hand, the above-mentioned progress focused on NaYF_4_ micro/nanocrystals instead of mesocrystals. Mesocrystals are three-dimensional (3D) order nanoparticles superstructures with unique properties and various potential applications as functional materials [35,36,37,38]. Nevertheless, the range of known mesocrystallines remains quite limited, in which few investigations to fluorine-containing compound mesocrystallines are available [39,40,41,42,43,44,45]. More recently, our group fabricated yttrium hydroxide fluoride mesocrystalline, as well as its Eu^3+^ doped analogue, by means of an additive-free hydrothermal procedure, which involved the reaction of Y(NO_3_)_3_, NaF, and NaOH aqueous solution without any organic additives [39]. Furthermore, we explored the preparation of rare-earths trifluoride mesocrystals by a solvothermal route involving IL 1-butyl-3-methylimidazolium hexafluorophosphate (BmimPF_6_) as the fluorine source in the presence of 1,4-butanediol [40]. However, no effort has been made to reveal the phase control related to rare-earths fluoride mesocrystallines. Herein, we present a facile, one-pot route called a polyol-mediated fluoride slow-releasing strategy for the rational control of pure phase α-NaYF_4_ mesocrystals. In spite of a higher temperature or/and higher doping level, cubic phase can be maintained.

## 2. Experimental Procedure

### 2.1. Chemicals and Materials

Analytical grade rare earth chlorides and/or nitrates (yttrium chloride hexahydrate, gadolinium chloride hexahydrate, ytterbium nitrate pentahydrate, and erbium nitrate pentahydrate, 99.9%) were provided by Aladdin Industrial Inc. Shanghai, China. NaNO_3_ (99.0%), 2,2′-oxydiethanol (99.0%, diethylene glycol, abbreviated as DEG), 1,2-ethanediol (99.0%), and ethanol (99.8%) were obtained from Sinopharm Chemical Reagent Company, Shanghai, China. 1-Butyl-3-methylimidazolium hexafluorophosphate (BminPF_6_, 99%) was purchased from Aldamas-beta Co., Shanghai, China. All of the reagents and solvents were directly used without further treatment.

### 2.2. Synthesis

NaYF_4_:Yb^3+^,Er^3+^(20/2 mol%) (abbreviated as NYF:Yb^3+^,Er^3+^ hereafter) and Gd^3+^ tri-doped NYF:Yb^3+^,Er^3+^(20/2 mol%) nanocrystals (NCs) were synthesized via a polyol-mediated solvothermal procedure. Here, we took the synthesis of NYF:Yb^3+^,Er^3+^ (20/2 mol%) as an example. The starting chemicals including NaNO_3_, yttrium chloride hexahydrate, ytterbium nitrate pentahydrate, and erbium nitrate pentahydrate in the stoichiometric ratio were well mixed with 1,2-ethanediol (or DEG) under stirring, to form solution. Thereafter, the solution was slowly added into a 25-mL polytetrafluoroethylene (PTFE) vial containing a proper amount of BminPF_6_ under vigorous stirring. The autoclave was sealed after vigorous stirring at room temperature for around 15 min, and then heated at 120 °C for 24 h. The final products were collected by centrifugation, and then washed sequentially using ethanol and H_2_O three times. After drying at 70 °C under dynamic vacuum for 24 h, an NYF:Yb^3+^,Er^3+^ sample was obtained. The synthetic procedure of Gd^3+^ tri-doped NYF:Yb^3+^,Er^3+^ (20/2 mol%) NCs was the same as that which was used to fabricate NYF:Yb^3+^,Er^3+^, except that the stoichiometric amount of gadolinium chloride hexahydrate was also added to 1,2-ethanediol (or DEG).

### 2.3. Characterization

The crystal structure and phase analysis were determined via X-ray diffraction (XRD) using a Bruker D8 Advanced X-ray diffractometer (Ni filtered, Cu K_α_ radiation, 40 kV and 40 mA) (Bruker, Billerica, MA, USA). The morphology the products were recorded on a transmission electron microscope (TEM, JEM-2010, JOEL Ltd., Tokyo, Japan) and a Hitachi S4800 field-emission scanning electron microscope (FE-SEM) (Hitachi Ltd., Tokyo, Japan). The selected area electron diffraction (SAED) pattern were characterized by the above-mentioned TEM (JEM-2010). An up-conversion fluorescence spectrum was obtained on an Edinburgh Instrument FLS920 phosphorimeter (Edinburgh Instruments Ltd., Livingston, UK) with a 980-nm laser diode Module (K98D08M-30mW, Changchun, China) as the excitation light source. The above-mentioned measurements were performed at room temperature from powder samples.

## 3. Results and Discussion

NaYF_4_-based mesocrystals were fabricated via solvothermal treatment of Na^+^, Y^3+^, and BminPF_6_ in the presence of viscous polyol-like diethylcol or 1,2-ethanediol. Apart from serving as solvent and complexant (i.e., bonding with Na^+^ and Y^3+^), polyol also acts as a stabilizer that limits particle growth and suppresses the α→β phase transition of NaYF_4_ [22,46]. BmimPF_6_ was chosen as a task-specific fluorine source (the reason why it was chosen as the fluorine source is given in the Supplementary Materials). The required fluoride anion (F^−^) was provided by BmimPF_6_ as a result of its slow decomposition and hydrolysis [23,45,47]. Even without additional water, BmimPF_6_ can hydrolyze with the aid of the trace water and hydration water molecules from yttrium chloride hexahydrate [45,48]. During the treatment procedure, PF_6_^−^ can slowly hydrolyze and then produce F^−^ through slowly increasing the temperature [45], as revealed in Equation (1):

PF_6_^−^ (IL) + H_2_O → PF_5_·H_2_O + F^−^ (1)

Therefore, this procedure was defined as a fluoride slow-release strategy, which involved fluoride releasing from BmimPF_6_ with the assistance of polyol [49].

**Figure 1:**
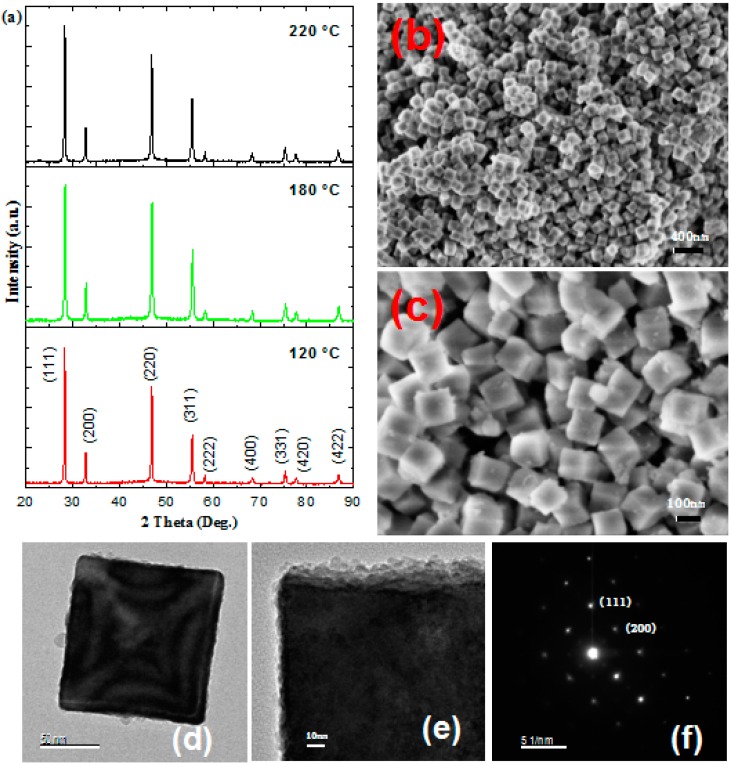
(**a**) X-ray diffraction (XRD) patterns of sodium yttrium fluoride (NYF):Yb^3+^Er^3+^ (20/2 mol%) sample at various solvothermal temperatures using diethylene glycol (DEG) as the reaction medium (all of the diffraction peaks are attributed to cubic-phase NaYF_4_), field-emission scanning electron microscope (FE-SEM) images ((**b**) low-magnification; (**c**) high-magnification), (**d**,**e**) TEM images, and (**f**) selected area electron diffraction (SAED) pattern of as-obtained NYF:Yb^3+^,Er^3+^ (20/2 mol%) submicrocrystals at 120 °C. (Note the nanoparticles aggregated to form submicrocubes).

**Figure 2:**
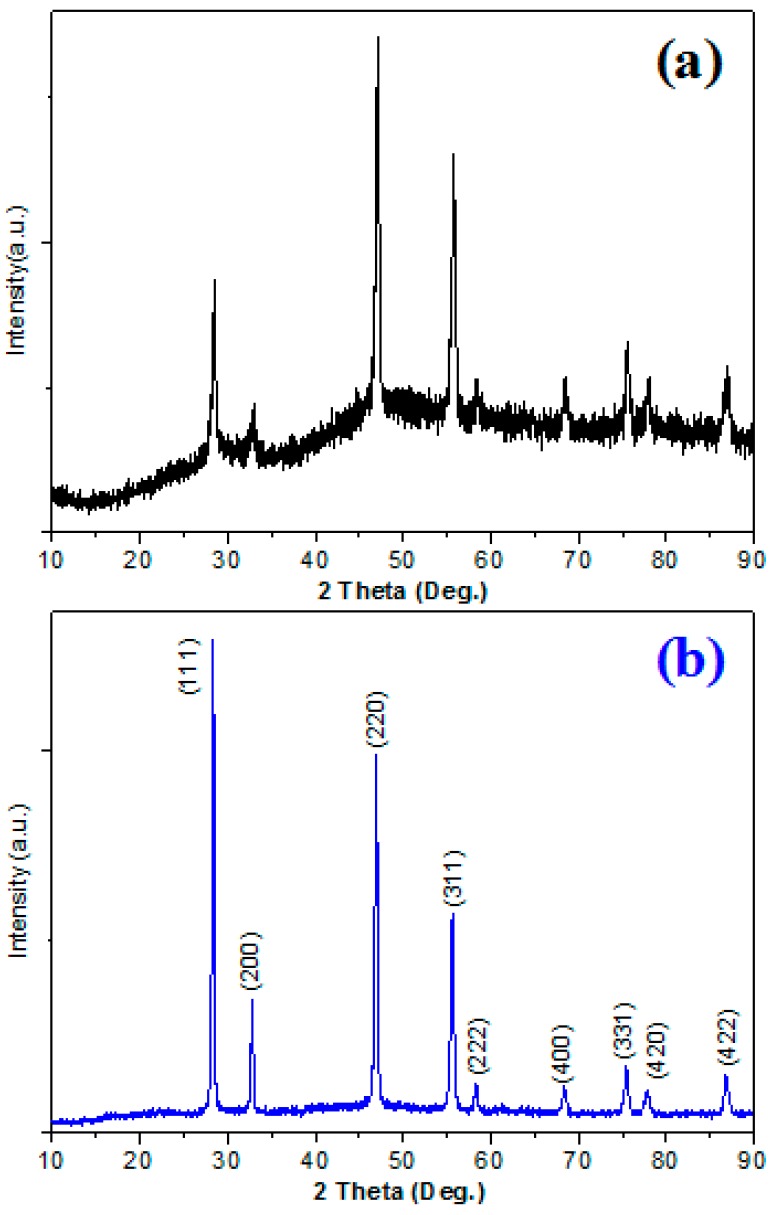
XRD patterns of (**a**) NaYF_4_ host, and (**b**) NYF:Yb^3+^,Er^3+^ (20/2 mol%) samples using 1,2-ethanediol as solvent.

Powder XRD patterns of a Yb^3+^-Er^3+^ co-doped and pure NaYF_4_ submicrocube in the case of DEG as the reaction medium are illustrated in Figure 1a and Figure 2a, respectively. All of the diffraction peaks matched the α-phase NaYF_4_ crystals (PDF No.77-2042), and no impurities were found. The sharp and narrow diffraction peaks revealled the highly crystallinity of these submicrocubes despite treatment at relatively low temperature (120 °C).

As exhibited in Figure 1b–d, all of the NYF:Yb^3+^,Er^3+^ submicrocrystals show cubic shapes and edge lengths of about 120 nm. Both FE-SEM and TEM photos illustrated their novel microstructure features, which are built from many nanoparticles and exhibited rough surfaces. A few nanoparticles were found attached on its surface (Figure 1e). Especially, the SAED pattern (Figure 1f) of a single NaYF_4_ cube shows sharp and periodic spots, revealing its noticeable single crystal-like feature. According to Cölfen et al. [35,36,37,38,39,40], the regular-shaped NaYF_4_ cubes actually belong to typical mesocrystals. The combination of a coarse surface pattern and the attachment of nanoparticles reveal that these mesocrystallines resulted from the self-assembling of nanoparticle subunits rather than the classic crystalline growth [18,35,36,37,38,39,40].

When DEG was replaced by 1,2-ethanediol, the product can also be indexed as pure-phase cubic NaYF_4_ crystal (Figure 2b). All of these results indicated that IL BmimPF_6_ in the presence of polyol also acts as a crystal-phase manipulator during the formation of NaYF_4_ [18].

For comparison, the preparation of NYF:Yb^3+^,Er^3+^ was also conducted through an LSS procedure using NH_4_F as the F^−^ source and the mixture of ethanol–H_2_O–oleic acid as the medium at 120 °C and 220 °C [11,21]. Figure S2 (see Supplementary Materials) revealed the XRD patterns of as-obtained NYF:Yb^3+^,Er^3+^ (20/2 mol%) at 120 °C and 220 °C. Obviously, the product obtained at the lower temperature can be ascribed to a pure α-phase NaYF_4_, as expected for NaYF_4_ synthesized under mild conditions [21,23,32]. However, in the case of higher temperature (220 °C), only β-phase NaYF_4_ was fabricated. These results demonstrated that promoting the reaction temperature can induce the α→β phase change of NaYF_4_, which is consistent with previous reports [9,10,11,12]. However, as shown in Figure 1a, even if the reaction temperature reached 220 °C, the as-prepared nanoparticles via the fluoride ions-slow-release procedure unambiguously remained in a pure cubic phase. In a word, regardless of the treatment temperature, an α-phase NaYF_4_ can be obtained by this slow-release strategy.

**Figure 3:**
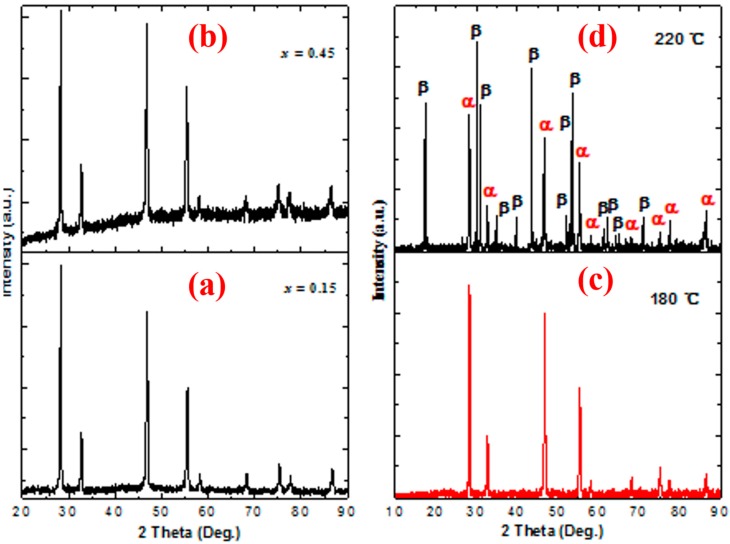
XRD patterns of Na (Y_0.78-*x*_Gd*_x_*), F_4_:Yb^3+^, and Er^3+^(20/2 mol%) samples with different tri-doping levels of Gd^3+^ ((**a**) *x =* 0.15, (**b**) *x =* 0.45), and Na (Y_0.48_Gd_0.30_)F_4_:Yb^3+^,Er^3+^(20/2 mol%) samples obtained at higher solvothermal temperatures ((**c**) 180 °C, (**d**) 220 °C; the symbol α and β represent cubic and hexagonal phases, respectively).

As mentioned above, without the tri-doping of Gd^3^^+^, the XRD pattern of the NYF:Yb^3+^,Er^3+^ (20/2 mol%) sample matched a cubic phase of NaYF_4_ (PDF No.77-2042). As for the NYF:Yb^3+^,Er^3+^ (20/2 mol%) sample, previous works revealed that introducing lanthanide ions (such as Gd^3+^) with a larger size than the Y^3^^+^ ion in the NaYF_4_ lattice not only induced an alteration from the α phase to the β phase, it also dominated the forming of pure β-phase NaYF_4_ NCs [1,20,50]. However, in this work, as revealed in Figure 3a, the pure cubic phase of NaYF_4_ remained when Gd^3^^+^ of 15 mol% was incorporated into host lattices. With the further increasing of the Gd^3^^+^ ion content (Figure 3b), no impurity diffraction peaks were found, showing the forming of a homogeneous solid solution, which is due to the small structural difference between the cubic-phase NaGdF_4_ and NaYF_4_. Obviously, phase transformation did not occur upon a higher-level doping of the dopant.

High-level doping usually leads to an α→β phase transition of NaYF_4_ in the LSS reaction system [1,20]. However, in present work, by using BminPF_6_ and polyol as the F^−^ source and reaction medium, respectively, as shown in Figure 3c, the as-synthesized submicrocubes remained in the cubic phase of NaYF_4_ in spite of higher total doping concentrations (52 mol%) as well as a higher solvothermal treatment temperature (180 °C). Even if the total doping contents were set as high as 52 mol%, and the treatment temperature simultaneously approached 220 °C (near to the work-limited temperature of the PTFE vial), the α-phase NaYF_4_ still existed in the products (see Figure 3d).

According to He et al. [51], the α→β phase change of NaYF_4_ can be attributed to the elevated content of F^−^ and the alteration to the reaction environment of Y^3^^+^ ions. In an LSS system involving oleic acid and a high active F^−^ source such as NH_4_F and NaF, it is found that the oleate anions are more likely to be combined with Y^3+^ in comparison with Na^+^ ions [4]. The interaction between oleate anions and Y^3+^ could effectively lower the energy barrier of the α→β phase transition [4]. Moreover, effective concentration of F^−^ ions was elevated, resulting from the rapid supply of F^−^. All of these could effectively promote the α→β phase transformation of NaYF_4_ [4,52,53]. Ultimately, β-phase NaYF_4_ was formed in an LSS system [9,10,11,12,21].

However, in the case of BminPF_6_ as the F^−^ source in the presence of polyol, the interaction between PF_6_^−^ ions and Y^3+^ was quite limited compared with the case of the above-mentioned LSS system [17,18]. In addition, noting the solubility product of NaYF_4_, *a_Na_a_Y_a_F_*^4^, the supersaturation degree (*a_Na_a_Y_a_F_*^4^/K_SP_) drastically varies with the content of fluoride ions, with an exponential relationship. Thus, the content of fluoride ions in a reactive system is of importance to the phase control of NaYF_4_ [15,19,33]. Herein, BmimPF_6_ slowly decomposes and hydrolyzes to create the required F^−^ during the elevation of the reaction temperature [23,45,47]. Therefore, BmimPF_6_ is a low-active F^−^ source relative to NH_4_F and NaF. Since the equilibrium constant of the hydrolyzed reaction (Equation (1)) is extremely small, the effective concentration of F^−^ ions was relatively low in the reaction system, which consequently results in a very slow precipitation with Na^+^ and Y^3+^ [45] (Equation (2)).

Na^+^ + Y^3+^ + 4F^−^ → NaYF_4_ ↓ (2)

In such circumstances, the supersaturation degree of the reaction system is not adequate to form the nuclei of the hexagonal phase NaYF_4_ [23]. Therefore, the formation of cubic phase NaYF_4_ was favored [51].

As mentioned above, polyol can complex with Na^+^ and Y^3+^. In addition, according to Chaumont et al. [54], PF_6_^−^ (IL) can coordinate with Y^3+^. Consequently, when BmimPF_6_ was uniformly dispersed in 1,2-ethanediol (or DEG) solution containing Na^+^ and Y^3+^ ions, these metal ions were believed to be simultaneously bonded by PF_6_^−^ anions as well as polyol [45]. In this case, Na^+^ and Y^3+^ ions were in the same shell surrounded by the imidazolium cation of BmimPF_6_ [23]. Upon thermal treatment, PF_6_^−^ slowly hydrolyzed and released F^−^, which was accompanied by forming NaYF_4_ nanosized grains; this can be evidenced by the nanoparticles that were attached on the surface of the as-obtained mesocrystals (Figure 1e). Subsequently, the polyol and IL co-stabilized nanoparticles aggregated to form NaYF_4_ mesocrystals, which possibly occurred through oriented attachment or mesoscale assembly processes due to the coexistence of a Coulombic force, van der Waals interaction, and hydrogen bonds in the system of polyol and BminPF_6_ [55,56]. Finally, it should be pointed out that the above-proposed forming course is only one of several possible mechanisms. Further studies about this issue are underway, and will be reported in future work.

**Figure 4:**
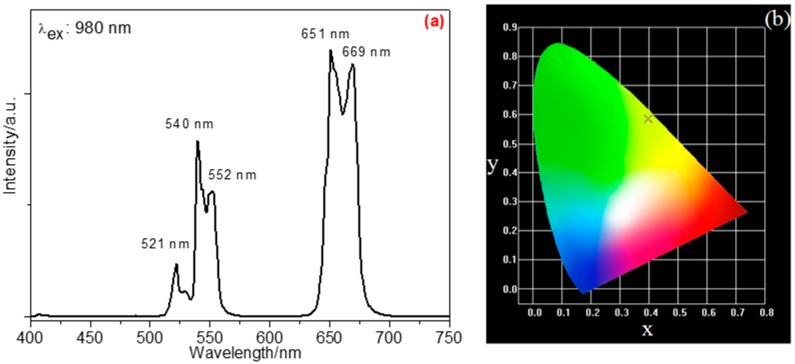
(**a**) Up-conversion luminescence spectrum at room temperature and (**b**) Commission Internationale de l’Eclairage (CIE) chromaticity diagram of NYF:Yb^3+^,Er^3+^(20/2 mol%) sample (λ_ex_: 980 nm).

Under the excitation of a 980-nm laser, α-phase NYF:Yb^3+^,Er^3+^ (20/2 mol%) mesocrystalline emitted bright yellow fluorescence, which demonstrated its photo functionality performance. The related luminescence spectrum is shown in Figure 4a. The green-emitting bands at about 521 nm and 540/552 nm are due to the ^2^H_11/2_ → ^4^I_15/2_ and ^4^S_3/2_ → ^4^I_15/2_ energy-level transitions of Er^3+^, respectively, while the red band at around 651/669 nm is assigned to the ^4^F_9/2_ → ^4^I_15/2_ transition of Er^3+^. The related Commission Internationale de l’Eclairage (CIE) coordinates are calculated as (*x* = 0.3984, *y* = 0.5854), which are situated in the region of yellowish light (point “×” in Figure 4b), revealing that it emitted yellowish light.

## 4. Conclusions

In summary, cubic-phase well-defined NaYF_4_ based photofunctional mesocrystallines were successfully prepared at relatively low temperature by using IL BmimPF_6_ and viscous polyol as the fluorine source and reaction medium, respectively. Combining slow-releasing fluoride via the decomposition and hydrolysis of fluorine-containing IL and the assistance of polyol, the formation of cubic-phase NaYF_4_ was favored, despite the higher treatment temperature or/and higher content of dopant. We believed that the key to the formation of uniform α-NaYF_4_-based mesocrystals is the use of fluorine-containing IL as a fluorine source as well as the existence of a polyalcohol. Our contribution offers a new alternative in constructing mesocrystal and other hierarchical nanostructured materials with an object phase under mild conditions.

## Supplementary Materials

The following are available online at http://www.mdpi.com/2079-4991/9/1/28/s1, Figure S1: XRD pattern of the sample using BmimBF_4_ as fluorine source (The bar represents the standard cards PDF#70-1935 for YF_3_), Figure S2: XRD patterns of NYF:Yb^3+^,Er^3+^ (20/2 mol%) NCs via LSS method at (a) 120 °C and (b) 220 °C (The bars in (a) and (b) represent the standard cards PDF#77-2042 and #PDF16-0334, respectively).
