# Supplementary material for: A Polyol-Mediated Fluoride Ions Slow-Releasing Strategy for the Phase-Controlled Synthesis of Photofunctional Mesocrystals
Source: Nanomaterials (Basel). 2018 Dec 26;9(1):28. doi: 10.3390/nano9010028 (PMC6359408; doi:10.3390/nano9010028)
Supplement: Supplementary file 1 [file nanomaterials-09-00028-s001.pdf]

nanomaterials

Communication

A Polyol-Mediated Fluoride Ions Slow-Releasing Strategy for the Phase-Controlled Synthesis of Photofunctional Mesocrystals

Xianghong He*, Yaheng Zhang, Yu Fu, Ning Lian, and Zhongchun Li

School of Chemistry and Environmental Engineering, Jiangsu University of Technology, Changzhou, Jiangsu 213001, China

* Correspondence: hexh@jsut.edu.cn; Tel.: +86-519-8695-3269

The reason why $BminPF_6$ rather than either 1-butyl-3-methylimidazolium tetrafluoroborate ($BmimBF_4$) or NH_4F was chosen as the fluorine source is as follows. Cui et al. found that a mixture of cubic and hexagonal phase $NaYF_4$ were obtained in a $BmimBF_4$-based system [1]. α-$NaYF_4$ based nano-/microcrystals with an irregular shape instead of mesocrystals were obtained via a solvothermal method using ployol such as glycol, diethylene glycol, or ethylene glycol (EG) as medium, as well as NH_4F or NaF as the fluorine source [2, 3]. Qu et al. reported that the sample prepared at low temperature (down to 80 ℃) via an EG-mediated solvothermal route using NH_4F as the fluoride source in the presence of trisodium citrate was made up of hexagonal $NaYF_4$ microspheres [4]. In this work, we failed to obtain $NaYF_4$ using $BmimBF_4$ as the fluoride source. As exhibited in Figure S1, the sample synthesized with $BmimBF_4$ was YF_3 nanocrystals with an orthorhombic phase structure (PDF No. 70-1935). Similar case was also found by Cui et al. [5] and Xu et al. [6]. Hence, we chose $BminPF_6$ rather than either $BmimBF_4$ or NH_4F as the fluorine source.

References

1. Meng He, Peng Huang, Chunlei Zhang, Feng Chen, Can Wang, Jiebing Ma, Rong He and Daxiang Cui, A general strategy for the synthesis of upconversion rare earth fluoride nanocrystals via a novel OA/ionic liquid two-phase system, Chem. Commun., 2011, 47(33): 9510-9512.
2. Wei Yu, Xiaojing Wang, Nan Chen, Guoping Du, and Weijun Gui, A strategy to prepare highly redispersible and strongly luminescent α-$NaYF_4$:Eu^{3+} hybrid nanostructures with multi-channel excitation, CrystEngComm, 2014, 16(15): 3214-3221.
3. Yang Wei, Fengqi Lu, Xinrong Zhang, Depu Chen, Polyol-mediated synthesis and luminescence of lanthanide-doped $NaYF_4$ nanocrystal upconversion phosphors, Journal of Alloys and Compounds 2008, 455: 376–384.
4. Xuesong Qu, Guohui Pan,Hyun Kyoung Yang, Yeqing Chen, Jong Won Chung, Byung Kee Moon, Byung Chun Choi,Jung Hyun Jeong, and Kiwan Jang, Low-temperature synthesis of luminescent and mesoporous β-$NaYF_4$ microspheres via polyol-mediated solvothermal route, RSC Adv., 2013, 3: 4763–4770.
5. Meng He, Peng Huang, Chunlei Zhang, Jiebing Ma, Rong He, and Daxiang Cui, Phase- and Size-Controllable Synthesis of Hexagonal Upconversion Rare-Earth Fluoride Nanocrystals through an Oleic Acid/Ionic Liquid Two-Phase System, Chemistry – A European Journal, 2012, 18(19): 5954-5969.
6. Congcong Mi, Zhenhuang Tian, Can Cao, Zhijia Wang, Chuanbin Mao,Shukun Xu, Novel Microwave-Assisted Solvothermal Synthesis of $NaYF_4$:Yb,Er Upconversion Nanoparticles and Their Application in Cancer Cell Imaging, Langmuir 2011, 27: 14632–14637.

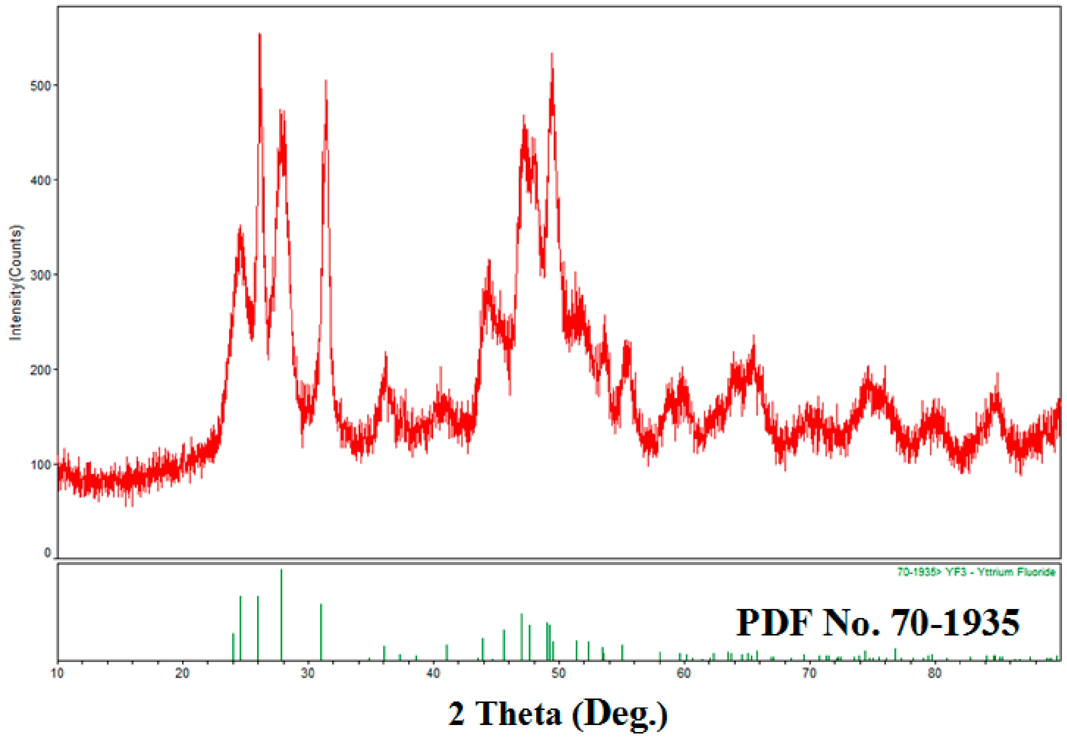

Figure S1. XRD pattern of the sample using $BmimBF_4$ as fluorine source (The bar represents the standard cards PDF#70-1935 for YF_3).

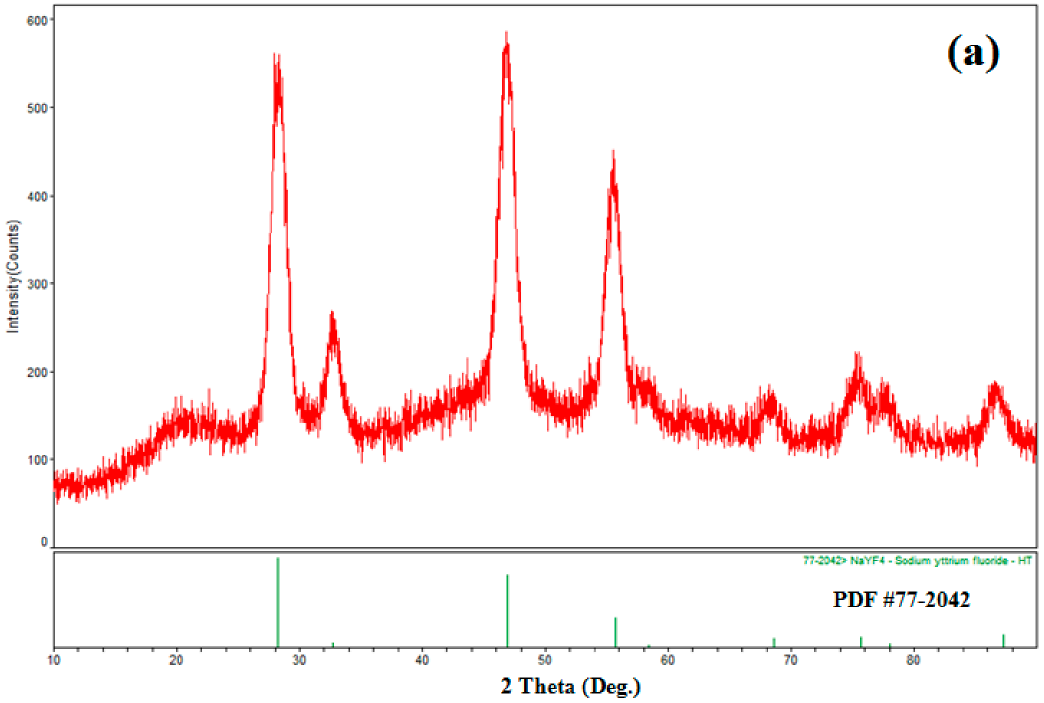

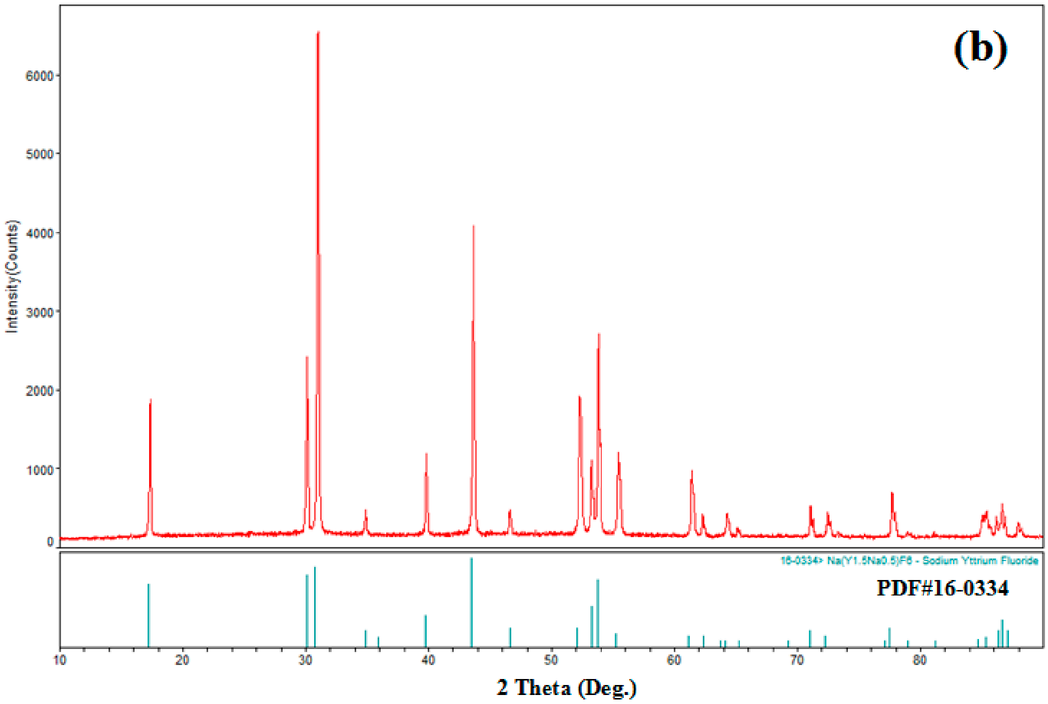

Figure S2. XRD patterns of NYF:Yb^{3+},Er^{3+} (20/2 mol%) NCs via a LSS method at (a) 120 °C and (b) 220 °C (The bars in (**a**) and (**b**) represent the standard cards PDF#77-2042 and #PDF16-0334, respectively).
